# A giant malignant phyllodes tumor of breast post mastectomy with metastasis to stomach manifesting as anemia: a case report and review of literature

**DOI:** 10.1186/s12893-020-00846-0

**Published:** 2020-08-15

**Authors:** Hui-Pu Liu, Wen-Yen Chang, Chin-Wen Hsu, Shan-Tao Chien, Zheng-Yi Huang, Wen-Ching Kung, Ping-Hung Liu

**Affiliations:** 1 Department of General Surgery, Kaohsiung Armed Forces General Hospital, No.2, Zhongzheng 1st Rd., Lingya Dist., Kaohsiung City, 802 Taiwan; 2 Department of Pathology, Kaohsiung Armed Forces General Hospital, No.2, Zhongzheng 1st Rd., Lingya Dist., Kaohsiung City, 802 Taiwan

**Keywords:** Malignant phyllodes tumor, Anemia, Stomach metastasis

## Abstract

**Background:**

Phyllodes tumors (PTs) are well known for local recurrence and progression. Less than 10% of these tumors grow larger than 10 cm. Distant metastases have been reported in up to 22% of malignant PTs, with most metastases being discovered in the lungs. PTs of the breast rarely metastasize to the gastrointestinal tract, and reported cases are scarce. To date, a review of the English literature revealed only 3 cases, including our case, of PTs metastasis to stomach.

**Case presentation:**

An 82-year-old female patient had 10-year-duration of palpable huge tumor on left breast which was in rapid growth in recent months. Total mastectomy of left breast was performed thereafter, and pathology diagnosis was malignant phyllodes tumor. Adjuvant radiotherapy was suggested while she declined out of personal reasons initially. For PTs recurred locally on left chest wall 2 months later, and excision of the recurrent PTs was performed. She, at length, completed adjuvant radiation therapy since then. Six months later, she was diagnosed of metastasis to stomach due to severe anemia with symptom of melena. Gastrostomy with tumor excision was performed for uncontrollable tumor bleeding.

**Conclusion:**

For PTs presenting as anemia without known etiologies, further studies are suggested to rule out possible gastrointestinal tract metastasis though such cases are extremely rare. Management of metastatic gastric tumor from PTs should be done on a case-to-case basis, surgical intervention may be needed if there is persistent active bleeding despite medical treatment. Adjuvant radiotherapy is recommended in borderline and malignant PTs with tumor-free margin < 1 cm and high-risk malignant tumors. Adjuvant chemotherapy or target therapy may be helpful for metastatic PTs. Molecular and genomic techniques may predict clinical outcomes of benign and borderline PTs more precisely.

## Background

Phyllodes tumors (PTs) account for 0.3 to 1% of all breast tumors and 2.5% of all fibroepithelial tumors of the breast [1]. Though PTs are similar to fibroadenomas in pathology, they have a double layered epithelial component surrounded by an increased stromal hypercellular component, forming leaf-like processes [2]. The median age of onset for PTs is 45 years [3]. PTs are classified by the World Health Organization (WHO) into benign, borderline, and malignant variants according to stromal cellularity, cellular atypia and pleomorphism, mitotic index, stromal overgrowth, tumor margin, and the presence or absence of heterologous differentiation [2].

The median size of PTs is about 4 cm. Those size larger than 10 cm are less than 10% of PTs, and the largest one so far is up to 50 × 50 cm reported by Islam et al. [4, 5] Approximately 10–15% of PTs are malignant, and only 10–26% of malignant PTs are found with metastasis [6–8].

## Case presentation

**Fig. 1:**
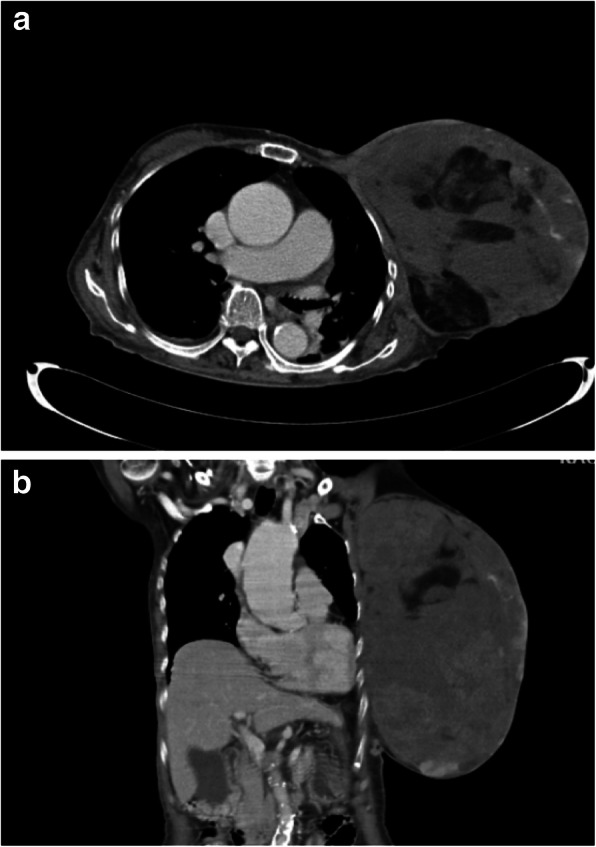
Axial view (**a**) and coronal view (**b**) of CT of chest-abdomen showed a huge breast tumor, L’t

**Fig. 2:**
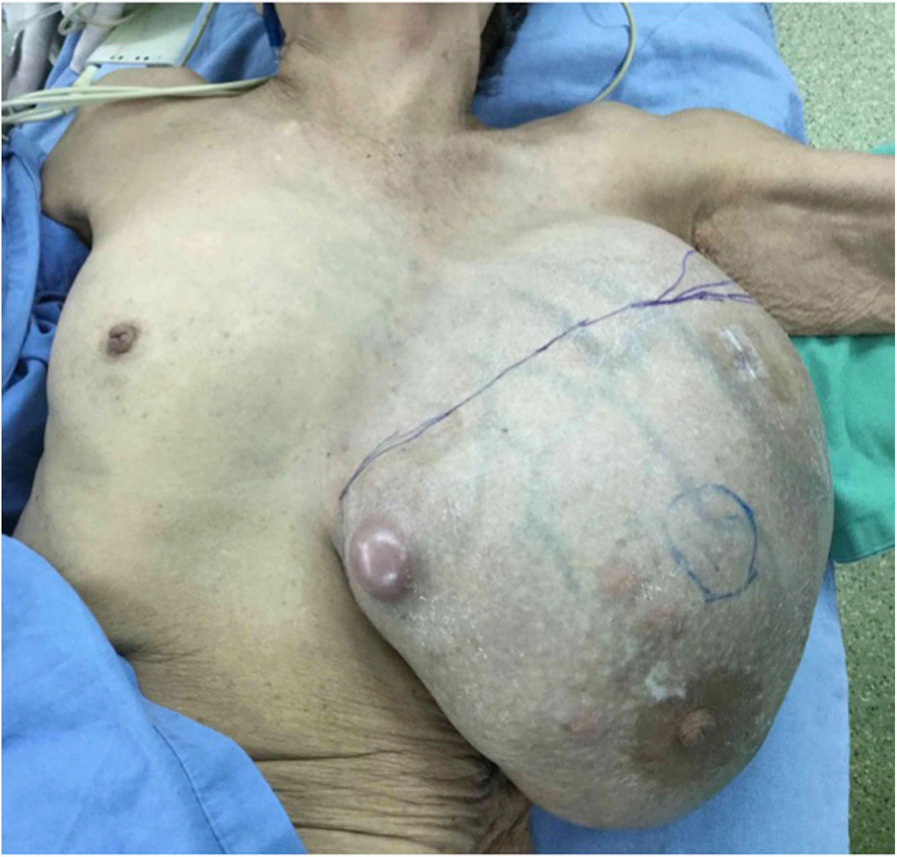
Shows a giant breast tumor, L’t

**Fig. 3:**
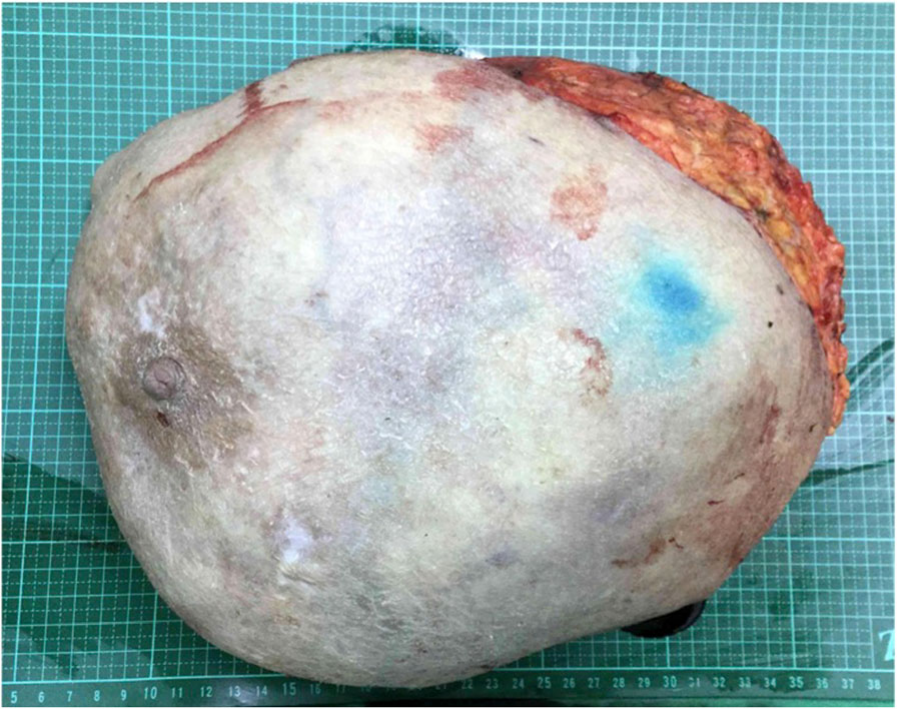
Show the gross picture of the excised breast tumor and its content

**Fig. 4:**
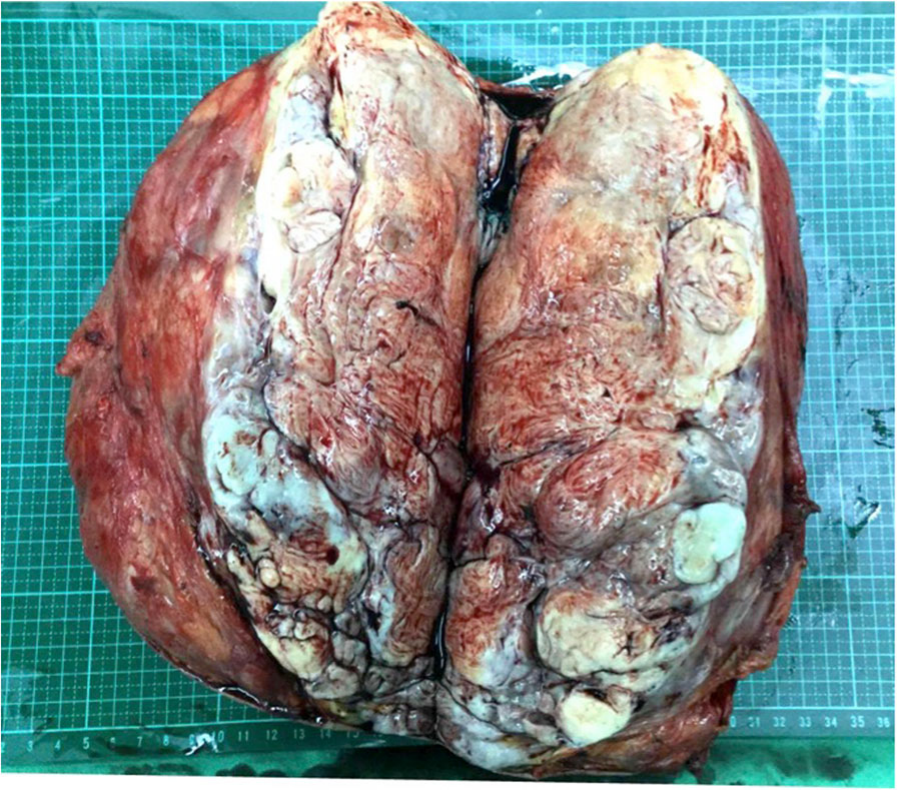
Show the gross picture of the excised breast tumor and its content

**Fig. 5:**
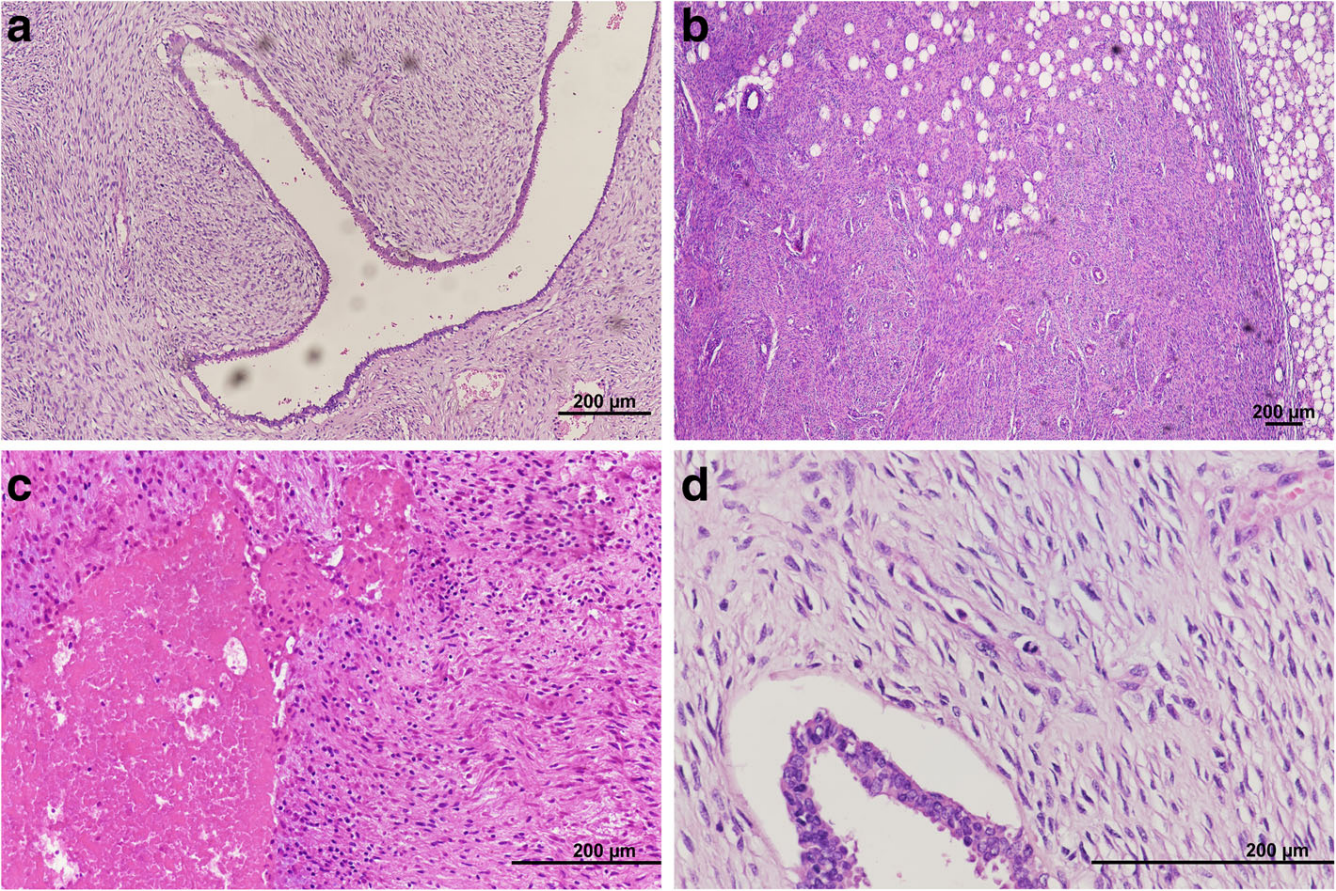
Microphotograph. **a** (H&E stain, × 100) diffuse hypercellularity tumor with compression of epithelium forming a “leaflike” pattern. **b** (H&E stain, × 40) permeative border of adipose tissue with stromal overgrowth. **c** (H&E stain, × 200) tumor necrosis. **d** (H&E stain, × 400) nuclear atypia and mitoses. (Using microscope: Nikon Ni-U. Digital Sight Camera: Nikon NS-Ri2. Camera and imaging software: NIS-Elements F Ver. 4.30.01)

An 82-year-old female patient had history of hypertension under medication for several years, and she presented to out-patient department in Feb., 2018 with 10-year-duration of palpable tumor on left breast which was in rapid growth in recent months. Otherwise, no dyspnea, bone pain, or headache were noted. Lab data showed WBC: 14500/μL, hemoglobin: 9.2 g/dL, alkaline phosphatase: 689 U/L, and CA-153: 65.6 U/ml. Computerized tomography of chest and abdomen showed no lung or liver metastasis (Fig. 1). Whole body bone scan showed no bony metastasis. Total mastectomy of left breast was performed thereafter (Figs. 2, 3 and 4). The excised specimen consisted of a large and firm oval mass partly covered by an elliptical skin, measuring 30 × 26 × 20 cm, weighed 4400 g. Pathology microphotograph revealed overgrowth of a diffused hypercellular tumor in stroma of breast tissue with nuclear atypia and mitoses indicating malignant phyllodes tumor of breast. High mitosis activity (10/10 HPF) and tumor necrosis were found (Fig. 5). However, the tumor free margin was less than 1 cm. Adjuvant radiotherapy was suggested while she declined out of personal reasons.

The patient had two local regional recurrent tumors over left chest wall 2 months later (Apr., 2018), and she underwent local excision then. She was subsequently followed by image guided radiotherapy with 5040 cGy/28 Frs since then. After completing whole course of radiotherapy, she was lost followed up.

**Fig. 6:**
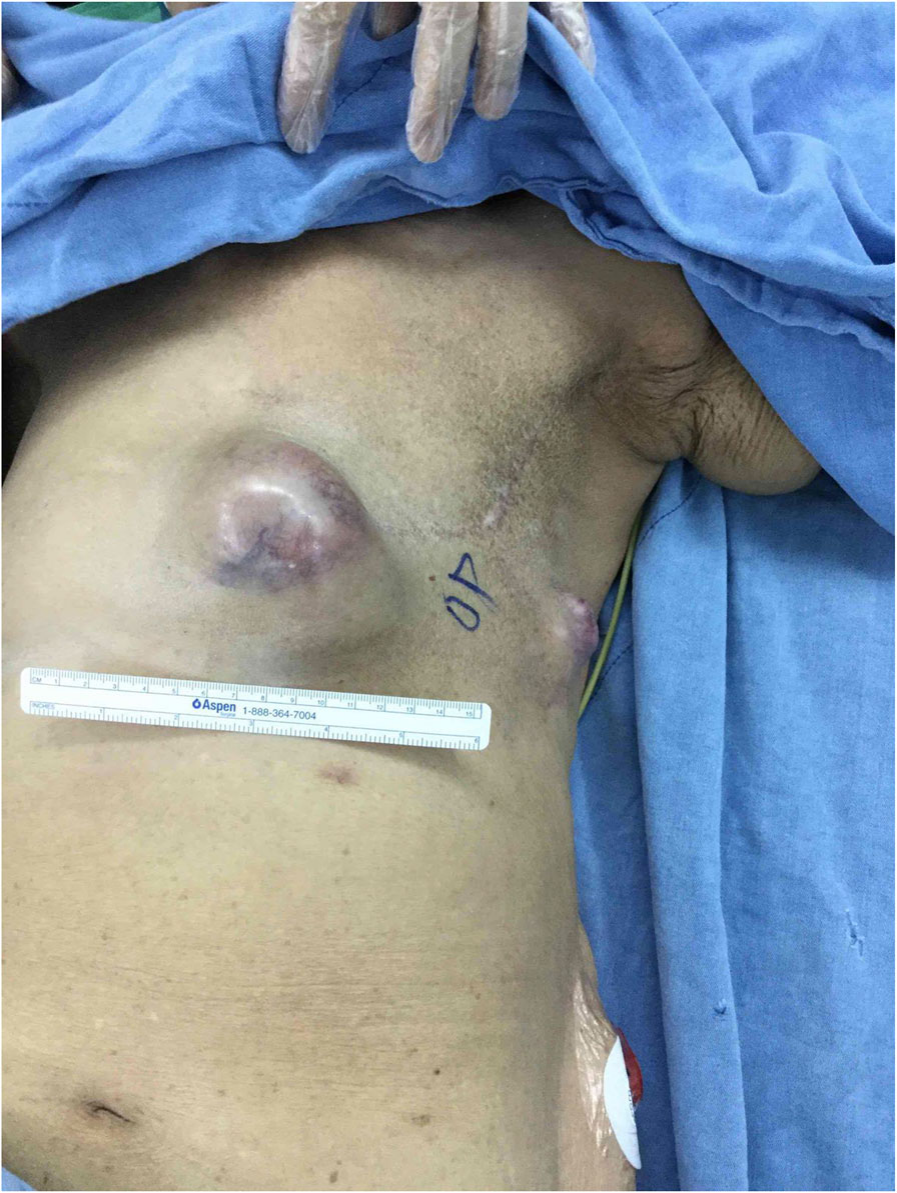
Showed recurrent tumors over left parasternal region and left sub-axillary region

**Fig. 7:**
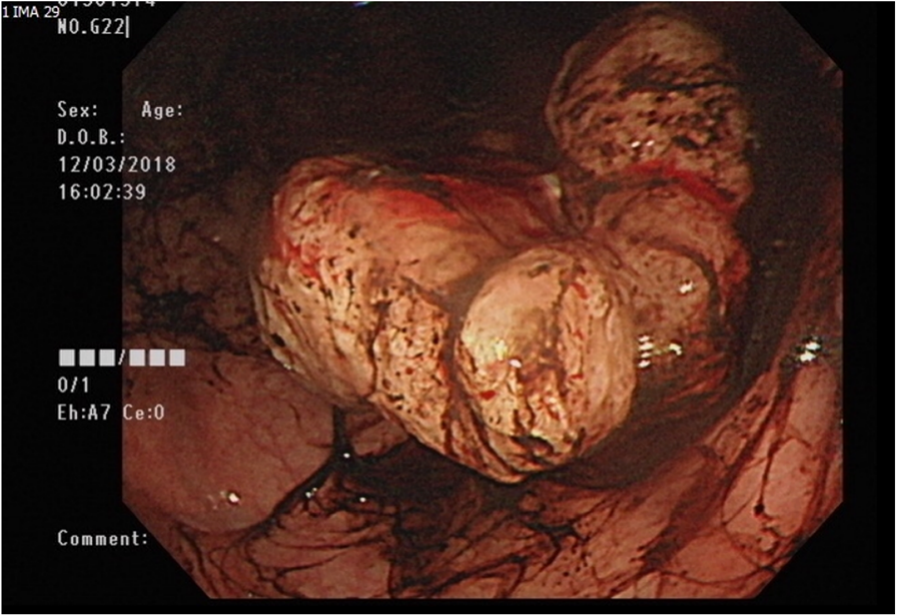
Showed an ulcerative gastric mass with punctate bleeding, Borrmann type 2

**Fig. 8:**
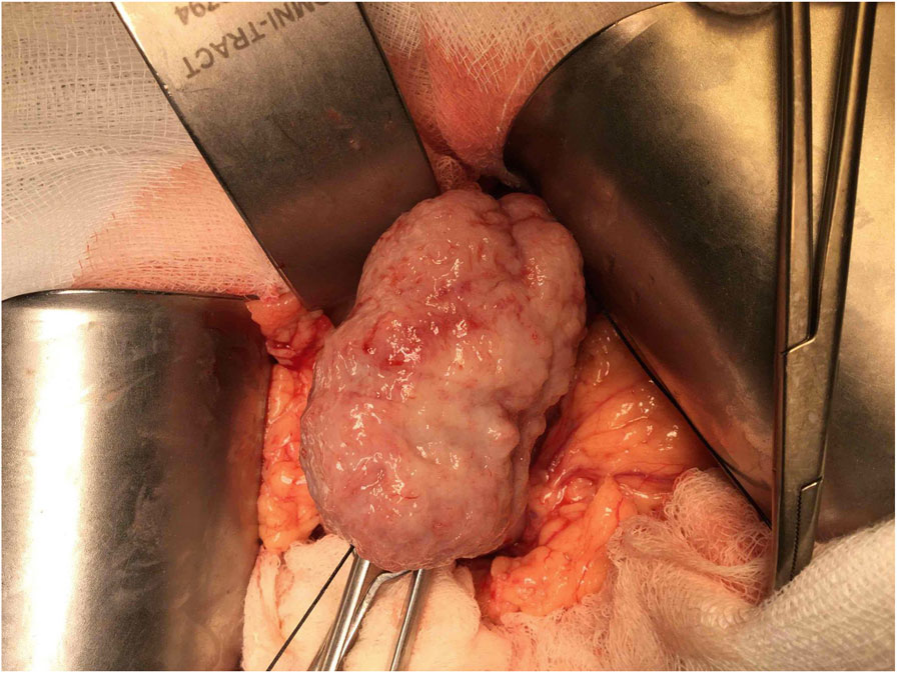
Showed a firmed, bulging tumor from gastric wall after gastrostomy

**Fig. 9:**
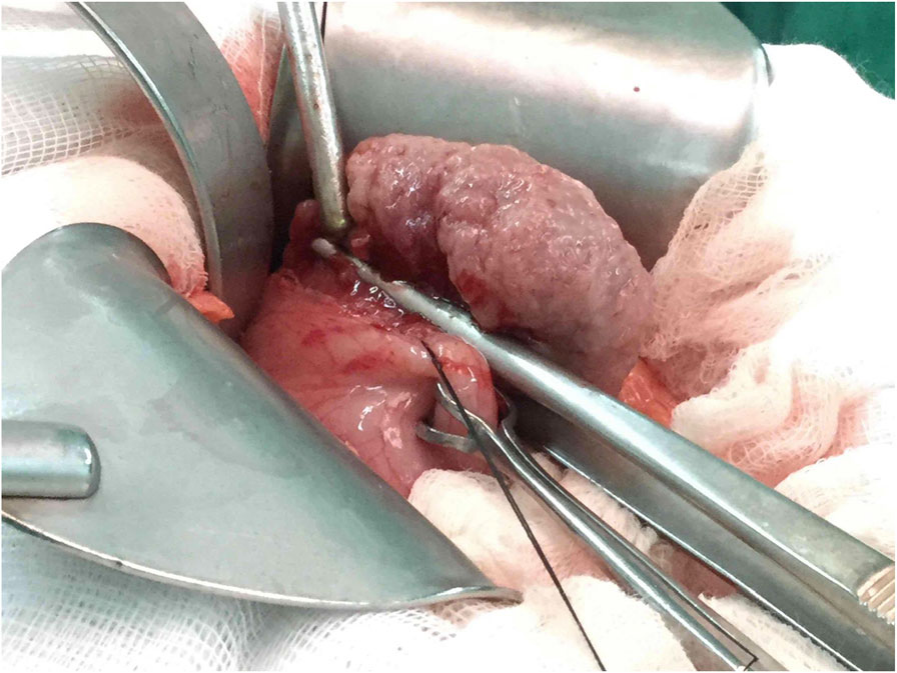
Excision of the tumor from the margin of gastric wall, and the specimen was confirmed of metastatic tumor from malignant phyllodes tumor of left breast. Tumor size: 5.8 × 3.5 × 2 cm

**Fig. 10:**
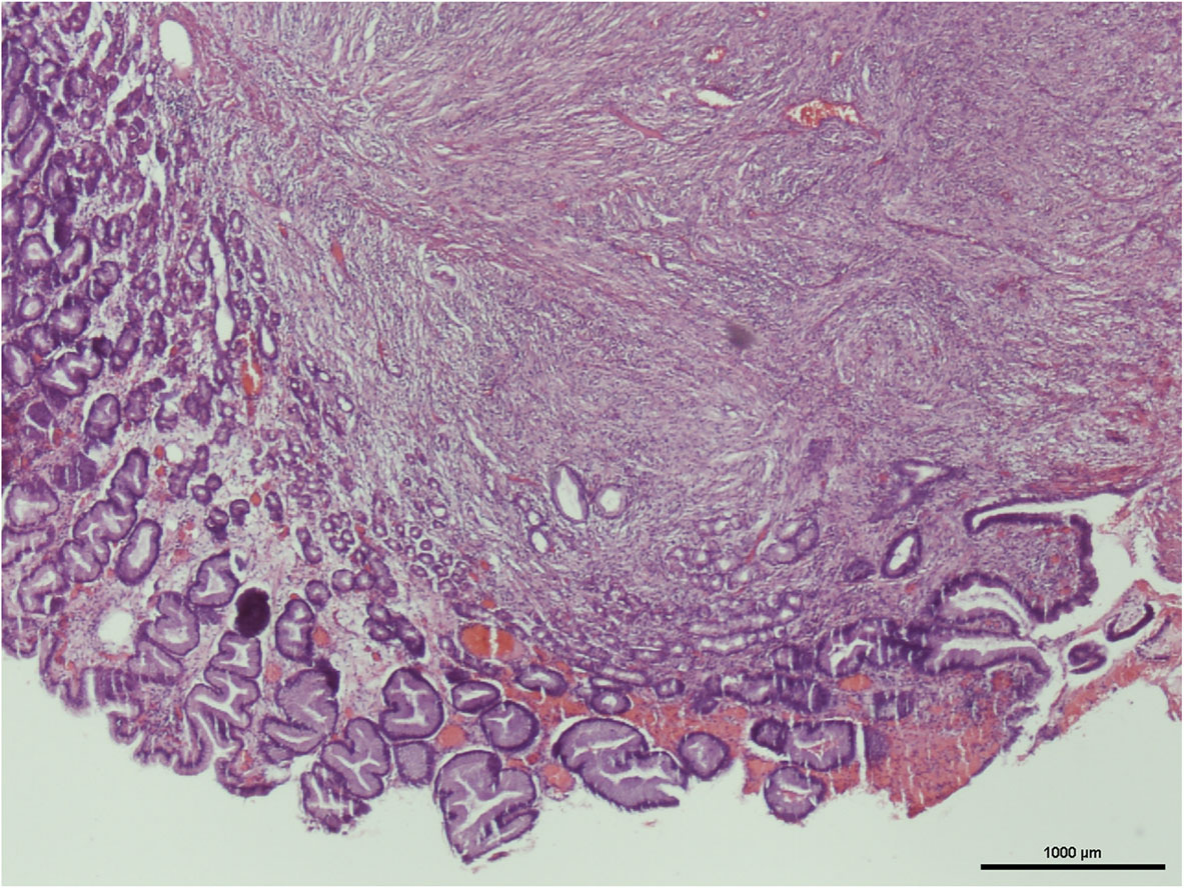
(H&E stain, × 40) showed a gastric tumor with stromal tissue (spindle cells) overgrowing disorderly, which is compatible with the metastatic phyllodes tumor from the breast. The immunochemical stain revealed all negative results for CD117, smooth muscle actin, and S100. (Using microscope: Nikon ECLIPSE 50i. Digital Sight Camera: Nikon DS-Fi1. Camera and imaging software: NIS-Elements F Ver. 4.30.01)

It’s not until 6 months later (Nov., 2018) her suffering from local recurrent tumors over left chest wall again that she came to out-patient department for help where scheduled excision of breast tumor was performed (Fig. 6). One tumor (7x5x5 cm) was located at left para-sternal region, and the other one (3.5x3x2.5 cm) was over left sub-axillary region. On 5th day after the operation, she had symptoms of passage of tarry stool and progressing weakness. On further inquiry, she has melena for 2 weeks. Laboratory data revealed severe anemia (decreasing hemoglobin level from 8.8 to 5.4 mg/dL in 1 week). Esophagogastroduodenoscopy (EGD) revealed a prominent protruding, ulcerofungating gastric tumor with punctate bleeding at fundus (Fig. 7). We performed gastrostomy with excision of the gastric tumor mentioned above due to refractory passage of tarry stool despite conservative treatment (Figs. 8 and 9). Metastatic tumor from malignant phyllodes tumor of left breast was confirmed by pathology (Fig. 10). After then, she didn’t experience any bleeding events anymore. Adjuvant chemotherapy was suggested while her family declined because of old age. During the hospital course, she was diagnosed of brain metastasis due to altered personality and decreased consciousness. Under hospice care in nursing home, she ultimately died 2 months later. (Mar., 2019).

## Discussion and conclusions

In malignant PTs, the rate of distant metastases have been reported in up to 22% [9]. The most metastases is found in the lungs [10], and some cases have metastasis to the kidney [11], duodenum [12], and pancreas [13]. Whereas, PTs of the breast metastasizing to the gastrointestinal tract are exceptionally rare. PTs metastasis to the stomach that presented as anemia were reported twice under medical treatment [14, 15]. However, our case underwent surgical intervention for uncontrollable gastric bleeding. To date, a review of the English literature revealed only 3 cases, including our case, of PTs metastasis to stomach.

In order to determine effective therapeutic plans for PTs, distinguishing between benign, borderline, and malignant is crucial in that there is significant variability in the interpretation of the criteria for each designation [16]. For diagnosing precisely in cases with malignant PTs, chest and abdomen should be screened with contrast enhanced CT at least even if the patient is asymptomatic [4].

According to Thind A., et al., a larger surgical margin of 1 cm or greater, compared with less than 1 cm, does not confer any statistically significant advantage as for local control, distant metastasis, or overall survival for borderline and malignant PTs [17]. Axillary dissection was not routinely performed since nodal metastases secondary to PT are very rare. In benign PTs, the traditional tumor-free surgical margin of 1 cm is not necessary.

Mastectomy related complications included wound infections requiring intravenous antibiotics or surgical debridement, wound dehiscence requiring reclosure, skin flap necrosis requiring surgical debridement, and hematomas or seromas at the mastectomy site requiring aspiration or drainage [18]. As for reducing seroma magnitude and duration, administration of fibrin glue may be useful [19].

Though adjuvant radiotherapy remains controversial (category 2B) for PTs according to National Comprehensive Cancer Network (NCCN) guidelines for the management of PTs (version. 5, 2020) [20, 21], use of radiotherapy has increased recently due to high risk of recurrence [22]. Adjuvant radiotherapy is recommended in borderline and malignant PTs with tumor-free margin < 1 cm [23]. For high-risk malignant tumors (higher grade, size > 5 cm), adjuvant radiotherapy is considered after R0 resection and recommended after R1 resection [24]. According to Chao et al., a meta-analysis with 696 patients enrolled in 17 studies, they found radiotherapy is effective in achieving local disease control and preventing metastasis [25]. Neoadjuvant radiotherapy for facilitating complete resection with definitive reconstruction to restore optimal functionality and patient satisfaction was also reported [26].

For metastatic PTs, some studies have reported promising results with administration of ifosfamide, doxorubicin and dacarbazine [27, 28]. According to Mitus et al., combination of doxorubicin plus cisplatin, cyclophosphamide, or ifosfamide could improve median survival in patients with metastatic phyllodes tumors [29]. Sunitinib, an oral multi-targeted tyrosine kinase inhibitor, was once noted in a case report providing a significant response of tumor reduction in patients with a metastatic phyllodes tumor [30].

The reliability of prediction of clinical outcomes based on morphological features (grade), even with clinical and radiological correlation, is poor. PTs are often misdiagnosed or mismanaged with dominant themes of under-diagnosis and under-treatment [31]. Using the molecular and genomic techniques to accurately predicts the prognosis will be imperative in the future. Mutation of MED12 and RARA, frequently observed in fibroadenomas and PTs, are highly associated with fibroepithelial tumorigenesis. Mutations in FLNA, SETD2 and KMT2D are suggested a role in driving phyllodes tumor development. Genomic landscape harbored *RB1*-truncating and *EGFR*-activating mutations, in addition to *MED12*, *RARA* and *FLNA* mutations, are consistent with the signature of a borderline or malignant phyllodes tumor [32].

In this case, she was diagnosed of distant metastasis to stomach after the symptoms of frequent melena and drop of hemoglobin level. However, she had anemia in the beginnings (hemoglobin level was in the range of 8 ~ 10 mg/dL) before total mastectomy. CT of chest-abdomen showed no overt evidence of distant metastasis while intraluminal lesions of gastrointestinal tract might be unclear in CT scan. Therefore, we suggest in those cases of PTs manifestating as anemia should be investigated for excluding possible either upper or lower gastrointestinal tract lesions. In this case, we didn’t perform molecular or genomic survey for predicting tumor behavior for the tumor itself was obviously malignant as for its tremendously large size and high mitotic rate. However, we recommended therapeutic plans for PTs in benign or borderline could be tailored via molecular or genomic profiles lest under-diagnosis or under-treatment.

In conclusion, for PTs presenting as anemia without known etiologies, further studies are suggested to rule out possible gastrointestinal tract metastasis though such cases are extremely rare. Management of metastatic gastric tumor from PTs should be done on a case-to-case basis, surgical intervention may be needed if persistent active bleeding despite medical treatment. Adjuvant radiotherapy is recommended in borderline and malignant PTs with tumor-free margin < 1 cm and high-risk malignant tumors. Adjuvant chemotherapy or target therapy may be helpful for metastatic PTs. Molecular and genomic techniques may predict clinical outcomes of benign and borderline PTs more precisely.

## Data Availability

The datasets supporting the conclusions of this article are included within the article.

## Abbreviations

PTs: Phyllodes tumors
CT: Computerized tomography
WHO: World Health Organization
EGD: Esophagogastroduodenoscopy
